# Impact of Structural Ageism on Greater Violence Against Older Persons: A Cross-National Study of 56 Countries

**DOI:** 10.1093/geroni/igab046.683

**Published:** 2021-12-17

**Authors:** E-Shien Chang, Joan Monin, Daniel Zelterman, Becca Levy

**Affiliations:** 1 Weill Cornell Medicine, New York City, New York, United States; 2 Yale School of Public Health, New Haven, Connecticut, United States; 3 Yale University, Woodbridge, Connecticut, United States

## Abstract

Violence directed against older persons is recognized as a global health problem. However, structural drivers for violence remain under-studied. This country-level ecological study aimed to examine a previously unexplored link between structural ageism and violence against older persons. Following extensive structural stigma literature, structural ageism consisted of two components: (1) discriminatory national policies related to older persons’ economic, social, civil, and political rights, gathered from global databases including UN, WHO, and others; and (2) societal-level prejudicial social norms against older persons, measured by negative attitudes toward older persons by the World Values Survey. Two components were z scored and combined such that higher score indicated greater structural ageism. Prevalence rates of violence per 100,000 persons aged 70 and over in each country were drawn from the Global Burden of Diseases Study. Final analysis included 56 countries, representing 63% of the world’s aging population aged 60 and over across all six WHO regions. As predicted, structural ageism was significantly associated with the prevalence rates of violence in multivariate models (β =205.7, SE=96.3, P=.03), after adjusting for country-level sociodemographic and health covariates. Three sets of sensitivity analyses supported the robustness of our findings. That is, structural ageism did not predict other types of violence and other types of prejudice did not predict violence against older persons. Public health and population-based violence prevention policies may benefit from a targeted approach that tackles the harmful effects of structural ageism.

